# Change by challenge: A common genetic basis behind childhood cognitive development and cognitive training

**DOI:** 10.1038/s41539-021-00096-6

**Published:** 2021-06-02

**Authors:** Bruno Sauce, John Wiedenhoeft, Nicholas Judd, Torkel Klingberg

**Affiliations:** 1 Department of Neuroscience, Karolinska Institute, Stockholm, Sweden; 2 Core Facility Medical Biometry and Statistical Bioinformatics, University Medical Center Göttingen, Göttingen, Germany

**Keywords:** Human behaviour, Intelligence

## Abstract

The interplay of genetic and environmental factors behind cognitive development has preoccupied multiple fields of science and sparked heated debates over the decades. Here we tested the hypothesis that developmental genes rely heavily on cognitive challenges—as opposed to natural maturation. Starting with a polygenic score (cogPGS) that previously explained variation in cognitive performance in adults, we estimated its effect in 344 children and adolescents (mean age of 12 years old, ranging from 6 to 25) who showed changes in working memory (WM) in two distinct samples: (1) a developmental sample showing significant WM gains after 2 years of typical, age-related development, and (2) a training sample showing significant, experimentally-induced WM gains after 25 days of an intense WM training. We found that the same genetic factor, cogPGS, significantly explained the amount of WM gain in both samples. And there was no interaction of cogPGS with sample, suggesting that those genetic factors are neutral to whether the WM gains came from development or training. These results represent evidence that cognitive challenges are a central piece in the gene-environment interplay during cognitive development. We believe our study sheds new light on previous findings of interindividual differences in education (rich-get-richer and compensation effects), brain plasticity in children, and the heritability increase of intelligence across the lifespan.

## Introduction

During childhood, cognitive abilities dramatically improve to make us who we are: persons capable of multiple academic, social, and professional activities. That process is incredibly complex—the interplay of genetic and environmental factors has preoccupied multiple fields of science and sparked heated debates over the decades^1,2^. Studies on the development of cognition have been surrounded by difficulties, and new advances are just now shedding light on mechanisms^3^.

It’s useful to start with the seemingly obvious fact that cognitive development differs from child to child. Some children develop their general cognition, such as reasoning, attention, and working memory (WM), at a rapid rate, while some others struggle far behind^4–6^. What explains these interindividual differences? We know that genetic inheritance must play a substantial role—decades of twin studies have shown cognition to be highly heritable in a multitude of populations and environmental contexts^7^. And recent studies show that genes can have powerful and complex interplays with the environment during development^4,8^.

How does gene-environment interplay come about? Many researchers favor the idea of natural maturation—during childhood, genes are the main drivers of development and need only a supporting role of common experiences available to almost every child, such as visual stimuli and social interactions (for reviews, see refs. ^2,9–11^). Like a train track, genes set the “default course” of development, while most environments serve as the coal to keep the train going (or, in the case of extremes environments, obstacles that can lag development). Under this maturation hypothesis (also known as experience-expectant), variation in cognitive change exists because some sets of genes code for brains with faster default maturation than others. In other words, children differ mostly because they have different default courses. In contrast, the cognitive challenge hypothesis (also known as skill learning or experience-dependent) proposes that the effect of developmental genes relies heavily on cognitive experiences^12–14^. Under that alternative explanation, variation in cognitive change exists because genes interplay with the many distinct (and idiosyncratic) cognitive challenges in children’s environments.

These two hypotheses have been well-studied for simpler traits in nonhuman species. There are traits that can be mostly explained by natural maturation, such a binocular vision in cats and heat resistance in fruit flies. In those traits, experience-expectant genes set a default course, and the environmental interplay with genes is expected and strictly planned by evolution^15^. For other traits, such as singing in songbirds and body growth in fish, there are large genetic variations that interplay with evolutionarily new and unexpected environments^15^. Those studies show there are multiple genes in nature that evolved to be flexible (or open-ended) towards the effect of particular experiences^16^.

However, for general cognitive abilities in humans, the picture is still inconclusive. We do not know if the interindividual differences in cognitive improvement in human children emerge mostly from experience-expectant genes (maturation hypothesis) or with flexible genes (cognitive challenges hypothesis). Due to practical reasons, it is extremely elusive to understand the interplay between genes and environments^4,17^. Two big problems have hindered progress: The first problem is that experiences are difficult to control or even measure accurately over years of development. So, studies on typical childhood development will always confound the cognitive challenge hypothesis with the maturation hypothesis—at first glance, any measured interindividual differences in cognitive development are as likely to arise from (flexible) genes interplaying with unknown cognitive experiences as they are to arise from (experience-expectant) genes via natural maturation. To contrast these hypotheses, we believe that developmental studies should be combined with intervention designs that create deliberate and trackable cognitive experiences. The second problem is that genetic studies with cognitive interventions have all looked at only a few candidate genes or DNA markers (e.g., refs. ^18–21^). General cognition is a complex polygenic trait, influenced by thousands of genetic regions, so those past studies cannot give us a full and reliable genetic picture.

Here we tested the cognitive challenge hypothesis for cognitive ability. To accomplish this, we combined genetic data from a longitudinal sample of typical childhood development and an intervention sample with intense, short-term (25-day-long) cognitive training. No study to date had such a design, partly due to conceptual quagmires (to our knowledge, we are the first to propose a study on gene-environment interplay in development by contrasting the role of a genetic set on the gains from cognitive training and on the changes in typical development) and partly due to lack of power. In the past, studies able to account for multiple genes needed to have hundreds of thousands of participants—an unrealistically large sample for experimental, cognitive interventions. Recently, that problem was partly overcome, thanks to the existence of reliable polygenic scores—indexes that put together thousands of genetic regions that alone would have extremely small predictive value. A large, genome-wide association study with 1.1 million people was able to estimate the effect of genetic regions on differences in cognitive performance, educational attainment, and mathematical ability^22^. With that information available, we were able to sum the reported effect sizes of all available genetic markers to create polygenic scores for cognitive performance (cogPGS) for each individual in our sample—providing our study with a much greater statistical power than typical in the past.

We focused on WM, a trait central to other general cognitive abilities, and responsible for the active maintenance and manipulation of information. High WM is associated with competence in reasoning and learning^23^, and benefit future school performance^24^, while low WM is associated with the inattentive symptoms of ADHD^25^. Recent studies have shown that structured WM training programs can have beneficial effects on an individual’s WM^26–29^, and suggests WM is, to some extent, malleable to experiences.

Our study rests on the following reasoning: if cogPGS can explain the variation in WM changes in both typical development and training, it suggests that both have genetic variation the interplays with cognitive experiences. This, we believe, represents a test of the cognitive challenge hypothesis—more specifically, the prediction that cogPGS explains the change of both development and training over time of cognition.

## Results

Our study included 344 children, adolescents, and young adults by combining a developmental and a training sample. The developmental sample (*n* = 160) was recruited to represent the general population, participants were between 6 and 25 years old and had their WM assessed twice with a 2-year interval. In the training sample (*n* = 184), participants were between 7 and 19 years old and completed an average of 24.7 days of WM training (SD = 1.06). In both samples, we measured WM performance by averaging standardized scores on a visuospatial and a verbal WM task.

We created polygenic scores for cognitive performance (cogPGS) using the SNP effect sizes from a multi-trait analysis that focused on a GWAS of cognitive performance and complemented by information from a GWAS on educational attainment and a GWAS on mathematical ability^22^.

### Change in WM

**Fig. 1:**
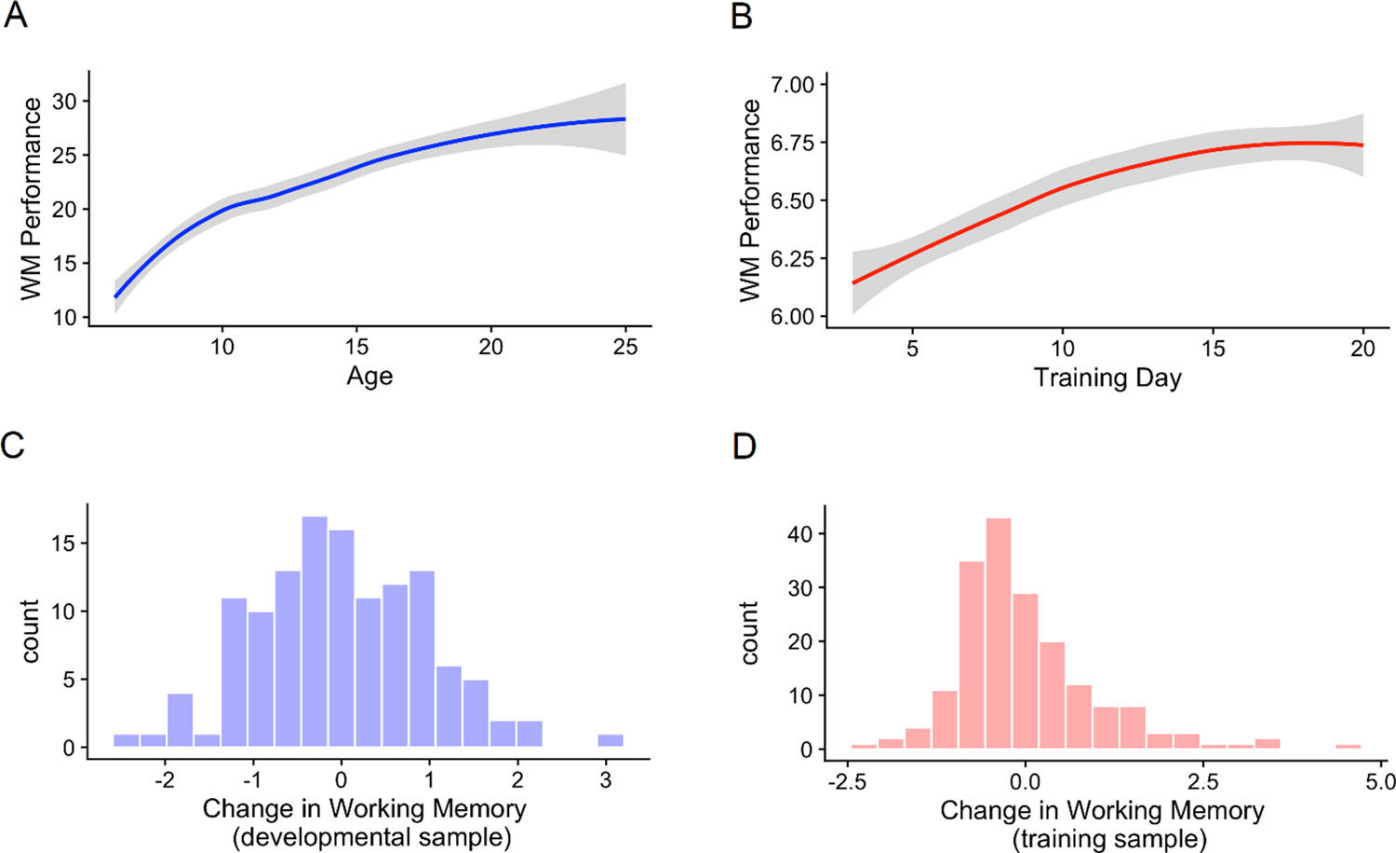
WM performance at different time points and the variability of WM change between individuals. **A** Baseline WM performance in the different age groups from the developmental sample. That variable is a combination of visuospatial and verbal working memory tasks and is total the number of correct trials given at the start of the study and averaged between the two tasks. Shades represent standard error. **B** WM performance on different days during cognitive training in the training sample. This daily WM performance is a combination of visuospatial and a verbal working memory tasks and represents the average level of the three successful trials with the highest level on each day and averaged between the two tasks. Shades represent the standard error of the mean. **C**, **D** Distribution of standardized change in WM per count of individuals in the developmental sample (after 2 years) and the training sample, respectively. The WM change variable is the subtracted baseline WM from the follow-up WM in each sample and then separately standardized (mean of zero and standard deviation of 1). Values of zero represent the mean change in each sample.

Figure 1A shows baseline WM performance in the developmental sample, showing an increase in capacity with age, with gradual flattening of development at older age-range. Baseline WM performance was subtracted from follow-up performance. This showed that, regardless of starting age, participants had a significant longitudinal increase in their WM after 2 years (β = 0.60, *p* < 0.001), with a mean increase of 14% (±2.5).

Figure 1B shows the average daily values in WM performance over the training duration in the training sample. Training led to a large average improvement in the performance of the trained WM tasks at the end of the intervention (β = 2.10, *p* < 0.001), with a mean increase of 34% (±1.5). Importantly, both samples showed large interindividual differences in the amount of WM change (Fig. 1C, D), which is the subtraction of baseline WM from the follow-up WM in each sample.

### Effect of age and gender on WM

To understand the effects on initial levels of WM, we created a model (here called “baseline WM model”) with baseline WM as the outcome and the predictors: sample (developmental and training), baseline age, gender, and cogPGS. Baseline WM was the performance on the first visit in the developmental sample, and maximum performance during days 2 and 3 of the training sample. As expected, we found that age affected the baseline levels of WM (β = 0.54, *p* < 0.001). There was no effect of gender (*p* = 0.44).

To understand the effects on the change in WM after 2 years (developmental sample) and after training (training sample), we subtracted baseline WM from the follow-up WM to obtain the variable WM change. We then used a general linear model (here called “WM change model”) with WM change as the outcome and the predictors: sample (developmental or training), baseline age, gender, cogPGS, age x sample, and cogPGS x sample. The model showed no effect of gender on the change in WM (*p* = 0.09). We also found that there was no main effect of age on the change in WM (*p* = 0.16), but an interaction of age with the type of sample (*p* = 0.04). For the developmental sample, younger participants changed more than older participants. This pattern was inversed in the training sample, with older participants improving more from the training.

### Effect of polygenic scores for cognitive performance on WM

Using the baseline WM model, we found that cogPGS significantly explained the baseline variation in WM (β = 0.10, *p* = 0.03). That translates into 1.0% of variance explained by cogPGS after accounting for the effect of gender and age differences. As described in Methods, note that this polygenic prediction is already controlled for population stratification, genotyping chip, and batch type (done during quality control of genotype data before obtaining the cogPGS), as well as gender and age (in the baseline WM model for prediction).

**Fig. 2:**
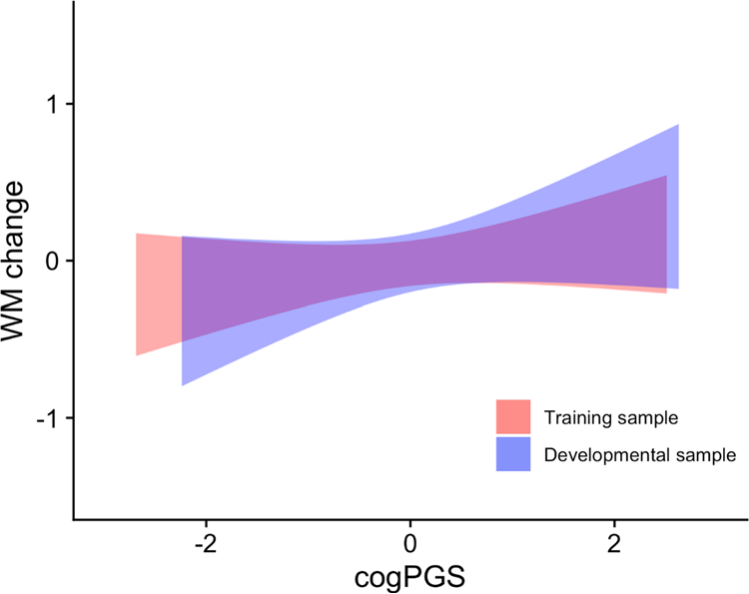
Regression of cogPGS on the change in WM for the training sample (red) and the developmental sample (blue). The WM change variable is the subtracted baseline WM from the follow-up WM in each sample and then separately standardized (mean of zero and standard deviation of 1). Values of zero represent the mean change in each sample. Values of cogPGS are also standardized to have a mean of zero and a standard deviation of one. Shades represent 95% confidence intervals.

Finally, we tested our main hypothesis: if cogPGS is related to interindividual differences in change in development and training and, if so, whether there are significant differences between training and development. The change in WM in both developmental and training samples can be seen in Fig. 2. The WM change model showed that cogPGS significantly explained interindividual differences in WM change (β = 0.15, *p* = 0.04), with 2.2% of variance explained after accounting for the effect of gender, age, sample, and the interactions. Importantly, there was no interaction of cogPGS with sample (*p* = 0.46), which suggests that the effect of cogPGS does not depend on the sample being developmental or training.

To check the consistency of our results, we reanalyzed our data with the following modifications: (1) Removing outliers with a WM change score beyond three standard deviations from the mean (five participants in total); (2) Using standardized age in each of the samples separately. In the original analyses, we used actual age—even though the two samples have around the same mean age, differences in standard deviation could have mattered. Reanalyses with these modifications (separately and combined) confirmed our main finding: that PGS has a significant association to change in WM (all *p* < 0.01), but there was no interaction between PGS and group.

## Discussion

The role of genetic factors in modulating the effect of experiences in cognitive development is a key question in developmental psychology, behavior genetics, and cognitive neuroscience. Here we showed that the amount of WM change in both a longitudinal sample of typical development and an intervention sample of cognitive training is in part explained by the same polygenic score (a set of genetic markers known to explain variation in cognitive performance at a single time point^22^). This genetic variation in plasticity, or malleability, is a pattern that in evolutionary biology has been considered a hallmark of flexible genes interplaying with new, unexpected environments^30,31^.

Our results here are evidence for the cognitive challenge hypothesis in cognitive development—more specifically, it supports the prediction that the genetic mechanisms of development should be partly shared with those of cognitive training. This implies that children’s cognitive abilities develop partly as a result of the cognitive challenges that they experience, much like a skill^12,32,33^. Our results are also evidence against purely natural maturation, in which cognitive development is genetically coded and with minimal influence of normal environmental variability. However, the two processes are not mutually exclusive and could coexist or dominate at different points during childhood.

One of the influential cognitive challenges during development might be schooling. Our finding here could thus explain the mechanism of why years of schooling, rather than chronological age, drives the development of WM^34^, as well as why education affects IQ^35^, and why twin studies show how environmental effects can be responsible for the change in cognitive function over time^36,37^.

For the developmental sample, younger participants exhibited more change (during the 2-year interval) than did older participants. This was expected and is in line with the literature on cognitive development, which increases with a rate that is inversely proportional to age, and approaches an asymptote in the early 20s (Fig. 1A)^38,39^. However, for the training sample, older participants improved more (during the 25-day interval) than younger participants. At first glance, this will look surprising. How could older children, whose brains are presumably less plastic, show more improvement? In the cognitive training literature, studies frequently find a Matthew effect (also known as the rich-get-richer effect or the magnification effect), where the participants with the highest initial cognitive values tend to be the ones getting the most gains from training^40–42^. There are a few potential reasons for the Matthew effect: (a) A child with higher WM will also focus better and therefore get more effective training and improve more; (b) The underlying mechanism why a child is high performing at baseline and why the child improves during an intervention might be partly identical. One such mechanism is highlighted in the present study: the same set of genes affect both development and response to an intervention; (c) Cognitive training programs often adapt to the performance of the user, so a high-performing child will get more challenged—resulting in a beneficial feedback loop. Important to our point here, this beneficial loop is not as strong and pronounced during many of the typical experiences/challenges in development, like a classroom (in fact, schools might plausibly show the opposite pattern, where teachers adapt and give more attention to the struggling students). This distinction in how “adaptive” the challenges are could be at the core of our contrasting finding in the effect of age in development versus training (at least when it comes to the “training” portion of developmental gains).

We believe our study also sheds light on the known increase in the heritability of intelligence during childhood development. Unlike many other traits, as we interact more with our environment over childhood, genetic effects seem to become more relevant to intelligence—heritability increases from 0.2 at 5 years of age to 0.6 by 16 years. Attempts to explain these results include models of gene-environment interplay—genetically endowed cognition influences one’s proximal environment and that environment in turn influences one’s cognition in continuous, reciprocal interactions, such as the multiplier theory^13^ and the transactional model^4^. Our finding here adds another line of evidence for these proposed models.

To our knowledge, only three other studies have measured the effect of any cognitive polygenic score on longitudinal change during development, and zero studies on cognitive training changes. Of those, two studies have failed to predict development from the cogPGS using as outcome measure either long-term memory^43^ or a broad cognitive measure (including decision making, pattern recognition, rapid visual processing, and WM)^44^. What could explain these contrasting findings? We believe this could be due to these polygenic scores for cognition having some degree of specificity. Our group recently looked at the cognitive change in typical development in a different sample^45^ and we identified the neural correlates with a polygenic score similar to our current cogPGS and using the same GWASs. That polygenic score was found to correlate with global surface area, and, even after correction for global effects, it was also associated with surface area in a single region located in the intraparietal cortex. This region is known to be linked to nonverbal, spatial cognitive abilities, including spatial attention, visuospatial WM, reasoning, and mathematics. This might explain why a cognitive polygenic score has a stronger association with the spatial abilities measured in our present study but not as strong with long-term memory (more strongly related to the medial temporal lobe) or with broad cognition (which includes a wider range of areas, including frontal cortical regions on decision making and possibly occipital areas involved in pattern recognition and rapid visual processing).

As was the case for the three studies mentioned above, our measure of cognitive change also has an important limitation: it is made of only two time points. Ideally, change should be estimated by three or more time points^46,47^. Two time points are still a viable way to measure change, but it comes with problems. In our analyses, we used the difference method to estimate change in WM (in other words, a subtraction between baseline and follow-up values), as it is likely the best method for our purposes here^48,49^. This method, however, always suffers from at least some regression to the mean—the size of this problem depends on how well the tasks are tapping into true performance (in other words, how much measurement error there is in each of the two time points). Because our WM measures were a composite of two tasks in both samples and were based on multiple trials (as described in more detail in Methods), that minimized the bias from regression to the mean.

All things considered—our results are of great theoretical importance to understand the genetics of flexible responses in cognitive development. We consider it a valuable piece in the elusive puzzle of gene-environment interplay in general cognition. In addition, given the critical role of WM and other general cognitive abilities in individual lives and societies, studies on the development of these traits could contribute to shape new avenues of research on training and plasticity, as well as help informing public policies on education and addressing at-risk groups with targeted interventions.

## Methods

Our study included a total of 344 children, adolescents, and young adults from the combination of two samples: a developmental sample and a training sample.

### Developmental sample

The developmental sample had 160 participants who were recruited using random sampling from a registry in Sweden and part of a longitudinal study of typical development. These individuals were in nine age groups (6, 8, 10, 12, 14, 16, 18, 20, and 25 years; mean age = 12.55, SD = 4.62), and have an equal gender distribution (78 females). More details about this sample in ref. ^21^. For the developmental sample, informed written consent to participate in the study was obtained from all participants over 18 years old and from the legal guardians of participants under 18.

### Cognitive training sample

In the training sample, we had 184 participants from Sweden who underwent cognitive training (described below) These individuals were between 7 and 19 years old at the time of training (mean age = 12.32, SD = 2.19), and have an equal gender distribution (86 females). More details in ref. ^19^. For the training sample, informed written consent to participate in the study was given by all participants older than 15 years and by all legal guardians for participants younger than 15 years.

The cognitive training in our training sample used the software Cogmed RM (Cogmed Systems, https://www.cogmed.com) developed by Torkel Klingberg^26,50^. Prior studies using exactly this method have shown significant improvements in WM when compared the improvement to passive control groups^51–53^ and also to active control groups^26,54,55^. Furthermore, studies with this method of training have related improvement to genetic polymorphism in candidate genes^19,20,56^.

The Cogmed training program consists of 12 different WM demanding tasks covering mostly visuospatial but also some verbal domains. Some of the tasks are changed during the training period to increase variability so that 8 of the 12 tasks are trained in each session.

As described in Holmes 2009^51^, each training task involved the temporary storage and manipulation of sequential visuospatial or verbal information or both. Three of the tasks involved the temporary storage of sequences of spoken verbal items, such as letters. These tasks tapped verbal short-term memory, although the simultaneous presentation of verbal information on the computer screen as it was spoken aloud in two of the tasks likely also tapped visuospatial short-term memory and WM. Two tasks involved the immediate serial recall of visuospatial information, such as a series of lamps that illuminated successively and, which the child attempted to recall in the correct order by clicking the appropriate location with the computer mouse. Verbal WM was tapped by two tasks, which involved the immediate recall of a sequence of digits in backward order. In one task the digits were spoken aloud at the same time as the corresponding numbers lit up on a keypad. Participants attempted to recall the sequence of digits in a backward sequence by clicking on the keypad. In a second task, the numbers were not displayed as they were spoken aloud. Three tasks required the processing and immediate serial recall of visuospatial information that was either moving around the screen during presentation and recall (e.g., asteroids that were continuously moving around the screen lit up one at a time and had to be remembered and recalled in the correct order) or moved spatial location between presentation and recall (e.g., lamps light up one at a time in a grid, the entire grid then rotates 90° and participants recall the order in which the lamps lit up, even though these are now in new positions). Motivational features in the program included positive verbal feedback, a display of the user’s best scores, and the accumulation of “energy” based on performance levels that was spent on a racing game completed after training each day. The racing game was included as a reward and did not tax WM.

All the training tasks had their difficulty level (number of items to be remembered) adapted on a trial-by-trial basis for each task. This was done according to a built-in algorithm that takes an individual’s previous performance into consideration. The adaptation allows for training to be performed at a level that is close to the capacity limit for each user. Participants completed an average of 24.7 training sessions (SD = 1.06) where each session lasted for an average of 36.5 min (SD = 8.8).

The study was approved by the regional ethical committees at Karolinska Institutet and the Karolinska University Hospital in Stockholm, Sweden.

### Measuring cognitive variables

We measured WM of all participants to create measures of baseline performance as well as the performance after training (in the case of the training sample) and after development (in the case of the developmental sample). WM in both samples was a combination of visuospatial WM and verbal WM.

In both samples, visuospatial WM was assessed using a similar task: a visuospatial grid task^57^ requiring remembering the location and order of dots displayed sequentially in a four-by-four grid on a computer screen. Verbal WM was also assessed in both samples using a similar task: the backward digit recall test, where numbers were read aloud to the participant who had to repeat them verbally in the reverse order. In the experimenter-led testing of the tasks, difficulty was increased by one level (number of items to be remembered) after at least two trials were correctly answered on one level. For the training group, difficulty was adjusted based on performance. Tasks terminated after three errors were committed on one level. The score used was the total number of correct trials.

For the developmental sample, we set the WM baseline as total the number of correct trials given at the start of the study and averaged between the visuospatial grid task and the backward digit recall test. WM performance after development was, again, the same measure but now between the two tasks given to the same participants after 2 years. For the Training sample, we set the WM baseline performance as the mean level of the three successful trials with the highest level on the visuospatial grid task and a verbal backward digit span task during days 2 and 3. WM performance after training was set as the mean level of the three successful trials with the highest level on a visuospatial grid task and a verbal backward digit span task during the two best training days. We used these measures for the training sample to keep the same standard used in a previous study^19^. As reported then, these two measures during training are also related to the WM performances at day 1 and day 20 (last day with complete data from all participants), with a correlation between mean day 1 WM performance and WM baseline of *r* = 0.875 and a correlation between day 20 WM performance and WM performance after training of *r* = 0.912^19^.

### Genotyping, quality control, and imputation

Blood and saliva samples were collected for genetic analyses in both samples, developmental and training. For the developmental samples, genomic DNA was extracted in a 96-wells format using the PureLink 96 genomic DNA kit K182104 (Invitrogen, United Kingdom). For the training sample, genomic DNA was extracted using OraGene OG-500 (DNA Genotek, Canada).

Genotyping was done in two batches. Batch 1 was genotyped on an Affymetrix Genome-Wide Human SNP Array 6.0. Liftover to human reference genome version hg19 was performed using liftOverPlink [Scott Ritchie: https://github.com/sritchie73/liftOverPlink]. Batch 2 was genotyped on an Illumina Infinium OmniExpressExome-8 v1.6 SNP array by SciLifeLab at Uppsala University.

After genotyping, quality control for individuals and markers was performed on both batches using the R package plinkQC [Meyer HV (2018) plinkQC: Genotype quality control in genetic association studies. 10.5281/zenodo.3373798] with PLINK v1.9b6^58^. This procedure also controlled for population stratification, genotyping chip, and batch type. In batch 1, there were 76 individuals and 424,323 variants that passed QC. In batch 2, that was true for 250 people and 543,103 variants. After quality control, we performed imputation of the remaining SNPs with IMPUTE2 v2.3.2^59^ using the 1000 Genome Project Phase 3 reference panel, at a window size of 5,000,000 bp, which yielded high concordance (Batch 1: 97.7%, Batch 2: 98.1%). Markers were filtered for existing RSIDs, and both datasets overlapped in 17,331,954 SNPs.

### Creating polygenic scores

We created polygenic scores for cognitive performance (here called “cogPGS”) for each participant using PRSice-2^60^. This was calculated by the sum of effect sizes of thousands of SNPs (weighted by how many of the effect alleles were present in each individual) that were discovered by a large genome-wide association study on educational attainment, mathematical ability, and general cognitive ability^22^. That study has available all effects sizes and *p* values of their SNPs on the website of the Social Science Genetics Association Consortium (https://www.thessgac.org/data).

We used the data available from a multi-trait analysis of GWAS^61^, which, in our case, represents a joint polygenic score focused on a GWAS of cognitive performance and complemented by information from a GWAS on educational attainment, a GWAS on the highest-level math class completed, and a GWAS on self-reported math ability (data called MTAG_CP by the consortium). This joint analysis is ideal because pairwise genetic correlations of these traits were high^22^. Furthermore, these GWAS had hundreds of thousands of individuals, and such a large sample size allows new studies to detect effects in samples of 100 individuals with 80% statistical power^22^.

For the creation of cogPGS in our samples, we performed clumping and pruning to remove nearby SNPs that are correlated with one another. The clumping sliding window was 250 kb, with the LD clumping set to *r*^2^ > 0.25. We included the weightings of all SNPs, regardless of their *p* value from the GWAS (*p* = 1.00 threshold). At the end of this process, we had 5255 SNPs included. We standardized the cogPGS to have a mean of zero and a standard deviation of one.

### Statistical analyses

To understand the effects on baseline levels of WM, we created a general linear model (here called “baseline WM model”) with baseline WM as the outcome and the predictors: sample (developmental and training), baseline age, gender, cogPGS. The model was: WM_baseline_ = Sample + Age_baseline_ + Gender + cogPGS. Baseline WM was standardized separately in each sample to have a mean of zero and a standard deviation of one.

Our main goal in the study was to test the independent influence of cogPGS on the change of WM. For that, we first subtracted baseline WM from the follow-up WM in each sample and then separately standardized them (mean of zero and standard deviation of 1) to obtain the variable WM change. We then used a general linear model (here called “WM change model”) with WM change as the outcome and the predictors: sample (developmental and training), baseline age, gender, cogPGS, age x sample, cogPGS x sample. The model was: WM_change_ = Sample + Age_baseline_ + Gender + cogPGS + cogPGS*Sample + Age_baseline_*Sample. For all analyses, we used univariate general linear models in SPSS version 26.

### Reporting Summary

Further information on research design is available in the Nature Research Reporting Summary linked to this article.

## Supplementary information

Reporting Summary

## Data Availability

The datasets generated during and/or analysed during the current study are available from the corresponding author on reasonable request.
