# Supplementary material for: Change by challenge: A common genetic basis behind childhood cognitive development and cognitive training
Source: NPJ Sci Learn. 2021 Jun 2;6:16. doi: 10.1038/s41539-021-00096-6 (PMC8172838; doi:10.1038/s41539-021-00096-6)
Supplement: Supplementary file 1 — Reporting Summary [file 41539_2021_96_MOESM1_ESM.pdf]

nature research

Corresponding author(s): Torkel Klingberg
Bruno Sauce

Last updated by author(s): Feb 17, 2021

Reporting Summary

Nature Research wishes to improve the reproducibility of the work that we publish. This form provides structure for consistency and transparency in reporting. For further information on Nature Research policies, see our Editorial Policies and the Editorial Policy Checklist.

Statistics

For all statistical analyses, confirm that the following items are present in the figure legend, table legend, main text, or Methods section.

n/a	Confirmed	
☐	☒	The exact sample size (n) for each experimental group/condition, given as a discrete number and unit of measurement
☐	☒	A statement on whether measurements were taken from distinct samples or whether the same sample was measured repeatedly
☐	☒	The statistical test(s) used AND whether they are one- or two-sided<br>*Only common tests should be described solely by name; describe more complex techniques in the Methods section.*
☐	☒	A description of all covariates tested
☐	☒	A description of any assumptions or corrections, such as tests of normality and adjustment for multiple comparisons
☐	☒	A full description of the statistical parameters including central tendency (e.g. means) or other basic estimates (e.g. regression coefficient) AND variation (e.g. standard deviation) or associated estimates of uncertainty (e.g. confidence intervals)
☐	☒	For null hypothesis testing, the test statistic (e.g. F, t, r) with confidence intervals, effect sizes, degrees of freedom and P value noted<br>*Give P values as exact values whenever suitable.*
☒	☐	For Bayesian analysis, information on the choice of priors and Markov chain Monte Carlo settings
☒	☐	For hierarchical and complex designs, identification of the appropriate level for tests and full reporting of outcomes
☐	☒	Estimates of effect sizes (e.g. Cohen's d, Pearson's r), indicating how they were calculated

Our web collection on statistics for biologists contains articles on many of the points above.

Software and code

Policy information about availability of computer code

Data collection	Cogmed RM (Cogmed Systems) https://www.cogmed.com
Data analysis	SPSS version 26

For manuscripts utilizing custom algorithms or software that are central to the research but not yet described in published literature, software must be made available to editors and reviewers. We strongly encourage code deposition in a community repository (e.g. GitHub). See the Nature Research guidelines for submitting code & software for further information.

Data

Policy information about availability of data

All manuscripts must include a data availability statement. This statement should provide the following information, where applicable:

- Accession codes, unique identifiers, or web links for publicly available datasets
- A list of figures that have associated raw data
- A description of any restrictions on data availability

The datasets generated during and/or analysed during the current study are available from the corresponding author on reasonable request.

Field-specific reporting

Please select the one below that is the best fit for your research. If you are not sure, read the appropriate sections before making your selection.

☐ Life sciences ☒ Behavioural & social sciences ☐ Ecological, evolutionary & environmental sciences

For a reference copy of the document with all sections, see nature.com/documents/nr-reporting-summary-flat.pdf

Behavioural & social sciences study design

All studies must disclose on these points even when the disclosure is negative.

Study description	Our study was a combination of two samples, each with their own study design: a developmental sample and a training sample. The developmental sample had a quantitative mixed design of cross-sectional and longitudinal, with different age groups having their cognition measured at baseline and 2 years later. The training sample had a quantitative experimental design with different age groups undergoing cognitive training and with cognitive measures taken before and after this intervention.
Research sample	Our study included a total of 344 children, adolescents, and young adults.<br>The developmental sample had 160 participants who were recruited using random sampling from a registry in Sweden. These individuals were in nine age groups (6, 8, 10, 12, 14, 16, 18, 20, and 25 years; mean age = 12.55, SD = 4.62), and have an equal gender distribution (78 females).<br>In the training sample, we had 184 participants from Sweden who underwent cognitive training. These individuals were between 7 and 19 years old at the time of training (mean age = 12.32, SD = 2.19), and have an equal gender distribution (86 females).
Sampling strategy	The developmental sample, we used participants whose parents agreed to participate based on a random sampling from the population registry in the town of Nynäshamn in Sweden.<br>For the training sample, information regarding the study was sent out via e-mail to, at that time, current customers of Cogmed WM training in Sweden, mostly consisting of schools.
Data collection	The data collection in our study used the software Cogmed RM (Cogmed Systems, https://www.cogmed.com).
Timing	*Indicate the start and stop dates of data collection. If there is a gap between collection periods, state the dates for each sample cohort.*
Data exclusions	All the data with cognitive and genetic information was included in the study.
Non-participation	For the developmental sample: Out of 1012 contacted individuals, 380 agreed to participate and 335 were eventually included for behavioral and genetic testing.<br>For the training sample: A responsible contact person at each school distributed these to individuals who were currently training or had previously completed training with Cogmed WM training program. A total of 1387 individual information envelopes were sent out. After consent was received, the questionnaires and a saliva self-collection kit were sent to the participants via mail. Completed questionnaires and saliva samples were returned by a total of 251 participants.
Randomization	N/A

Reporting for specific materials, systems and methods

We require information from authors about some types of materials, experimental systems and methods used in many studies. Here, indicate whether each material, system or method listed is relevant to your study. If you are not sure if a list item applies to your research, read the appropriate section before selecting a response.

Materials & experimental systems

n/a	Involved in the study	
☒	☐	Antibodies
☒	☐	Eukaryotic cell lines
☒	☐	Palaeontology and archaeology
☒	☐	Animals and other organisms
☐	☒	Human research participants
☒	☐	Clinical data
☒	☐	Dual use research of concern

Methods

n/a	Involved in the study	
☒	☐	ChIP-seq
☒	☐	Flow cytometry
☒	☐	MRI-based neuroimaging

Human research participants

Policy information about studies involving human research participants

Population characteristics	See above.
Recruitment	See above.
Ethics oversight	The study was approved by the regional ethical committees at Karolinska Institutet and the Karolinska University Hospital in Stockholm, Sweden.

Note that full information on the approval of the study protocol must also be provided in the manuscript.
